# Effect of Curcumin on the Increase in Hepatic or Brain Phosphatidylcholine Hydroperoxide Levels in Mice after Consumption of Excessive Alcohol

**DOI:** 10.1155/2013/242671

**Published:** 2013-03-28

**Authors:** Chang Won Pyun, Kyu-Ho Han, Go Eun Hong, Chi Ho Lee

**Affiliations:** ^1^Department of Food Science and Biotechnology of Animal Resources, College of Animal Bioscience and Biotechnology, Konkuk University, Seoul 143-701, Republic of Korea; ^2^Department of Food Science, Obihiro University of Agriculture and Veterinary Medicine, Obihiro, Hokkaido 080-8555, Japan

## Abstract

Curcumin is a bright yellow compound found in *Curcuma longa* L., a member of the family Zingiberaceae. In the present study, we determined whether curcumin protects against oxidative stress in liver and brain in mice fed excessive alcohol. BALB/c mice were administered 20% alcohol (16 g/kg body weight) with or without curcumin (0.016% in diet) for 12 weeks. The concentrations of phosphatidylcholine hydroperoxide (PC-OOH) in liver and brain samples were determined using chemiluminescence high-performance liquid chromatography. Mice treated with ethanol and curcumin significantly (*P* < 0.05) showed a lower hepatic PC-OOH level compared to mice treated with only ethanol. However, there was no significant difference in the brain PC-OOH level among all mice. Our finding indicates that the dosage of alcohol might increase the lipid peroxide level of liver but not of brain, and daily curcumin consumption might be protective for liver against alcohol-related oxidative stress in mice.

## 1. Introduction

The generation of reactive oxygen species (ROS) by alcohol consumption increases oxidative stress in the body [1]. Excessive alcohol consumption causes various diseases such as alcoholic fatty liver, hepatitis, and cirrhosis [2]. Furthermore, chronic exposure to alcohol-induced oxidative stress may promote carcinogenesis of liver tissue leading to cancer [1, 3]. In addition, dementia is an another major alcohol-related syndrome [4]. Although the mechanisms underlying alcohol-related brain disease and Alzheimer' disease are different, the affected part of the brain and the associated symptoms are similar [5, 6]. Oxidative damage is an early feature of Alzheimer' disease in mild cognitive impairment brains [7]. It might be possible that the oxidative stress might also affect an alcohol-related brain disease.

**Figure 1:**
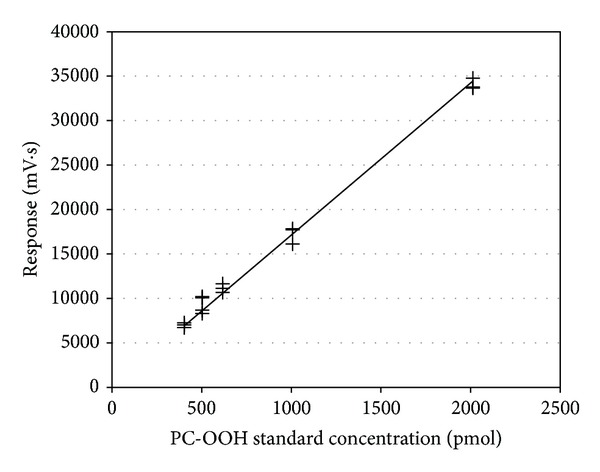
Calibration curve for phosphatidylcholine hydroperoxide (PC-OOH) obtained using the chemiluminescence high-performance liquid chromatography system.

Lipid peroxide in tissues has been used as a tool for impaired function of tissue or cell biomembrane [8–10]. Particulary, phosphatidylcholine (PC) is a major constituent of cell membranes, and the form of its hydroperoxide, phosphatidylcholine hydroperoxide (PC-OOH), provides a very sensitive and specific marker for oxidative stress in *in vivo* studies [11–13]. For example, the method for measuring PC-OOH level in animal tissue has been developed by using chemiluminescence analyzer-high-performance liquid chromatography (CL-HPLC) system and that has proven useful in many studies (Figure 1) [11]. Indeed, PC-OOH level in plasma or hepatic has been suggested to be available for a hepatic damage indicator [12]. Furthermore, Adachi et al. [13] found that alcohol consumption increases a plasma PC-OOH level, which might be also considered as a marker of oxidative stress caused by alcohol consumption.

Turmeric (*Curcuma longa* L.) is a popular spice in India, and curcumin is bright yellow pigment found in turmeric. It is a member of the family Zingiberaceae and has been used as a folk remedy in some cultures. The positive effects of purified curcuminoid on human diseases have been studied, including cancer [14], inflammatory [15], and Alzheimer's disease [16, 17]. For example, *in vitro* studies indicate that curcumin protects cells and tissues against oxidative stress by inhibiting generation of ROS [18–20]. In particular, Thong-Ngam et al. [21] reported that curcumin consumption protects liver against alcohol-induced oxidative stress through inhibition of NF-*κ*B activation. However, no information is available for the effect of dietary curcumin on alcohol-related PC-OOH level as oxidative stress maker in the liver and brain in mice.

Therefore, the present study was aimed to investigate the effect of curcumin on alcohol-induced oxidative stress by measuring PC-OOH level in liver and brain of mice. Additionally, we studied the effect of curcumin on alcohol-altered lipid composition in liver and brain.

## 2. Materials and Methods

### 2.1. Animals and Diet

8-week-old male BALB/c mice were purchased from Central Lab Animal Inc. (Seoul, Korea). All mice were daily fed a 4.5 g of AIN-93G modified diet [22] (in g/kg): 239 casein, 185 corn starch, 60 sucrose, 158 dextrose, 60 cellulose, 30 soybean oil, 207 lard, 42 mineral mixture, 12 vitamin mixture, 4 L-cystine, 3 choline bitartrate, and 0.14 tert-butyl hydroquinone. The diet composition was designed by the previous research [21, 23]. The mice were randomly divided into 3 groups (*n* = 5) as follows. ALC group was orally administered 20% ethanol daily, CUR group was orally fed a 0.016% curcumin (Sigma-Aldrich, Saint Louis, MO, USA) in diet after administering 20% ethanol [24, 25], and CON group was administered a volume of saline (0.85% sodium chloride) as equal to that of the alcohol administered to the other 2 groups. The dose of administered alcohol was 16 g/kg body weight to induce alcoholic diseases for 12 weeks, which was based on a long-term consumption as previous studies [26]. Mice were kept in house maintained a 12 h light/12 h dark cycle with 60% humidity and 25°C. After 12 weeks, the mice were anesthetized by intraperitoneal injection of Zoletil (Zoletil 50, Virbac, Carros, France), blood was collected from the abdominal aorta, and livers and brains were quickly removed and weighed before freezing for storage at −80°C.

### 2.2. Total Lipids, Triglyceride, and Cholesterol

Total lipid in the liver or brain was extracted by a modified method of Folch et al. [27]. First, 300–400 mg of tissue sample was homogenized with 2 mL of saline using a Teflon-glass overhead stirrer, and the homogenate was transferred to a conical tube. Next, 4 mL of chloroform-methanol solution (2 : 1, v/v) containing 0.002% butylated hydroxytoluene was added, and the solution was mixed vigorously using a vortex mixer for 1 min, and then centrifuged at 3000 ×g for 10 min. The lower layer was collected and transferred to another conical tube. The extraction process was repeated 3 times, and the lipid content was then determined gravimetrically after completely evaporating the chloroform with a stream of nitrogen gas. The extract was mixed with 2 mL of chloroform containing 2% triton X-100, and the organic phase was evaporated with stream of nitrogen gas, and the residue was diluted with 2 mL of distilled water. This solution was used for triglyceride (TG) and total cholesterol (TC) using commercial assay kit (Asan Pharmaceutical Co. Ltd., Seoul, Korea).

### 2.3. PC-OOH Standard

**Figure 2:**
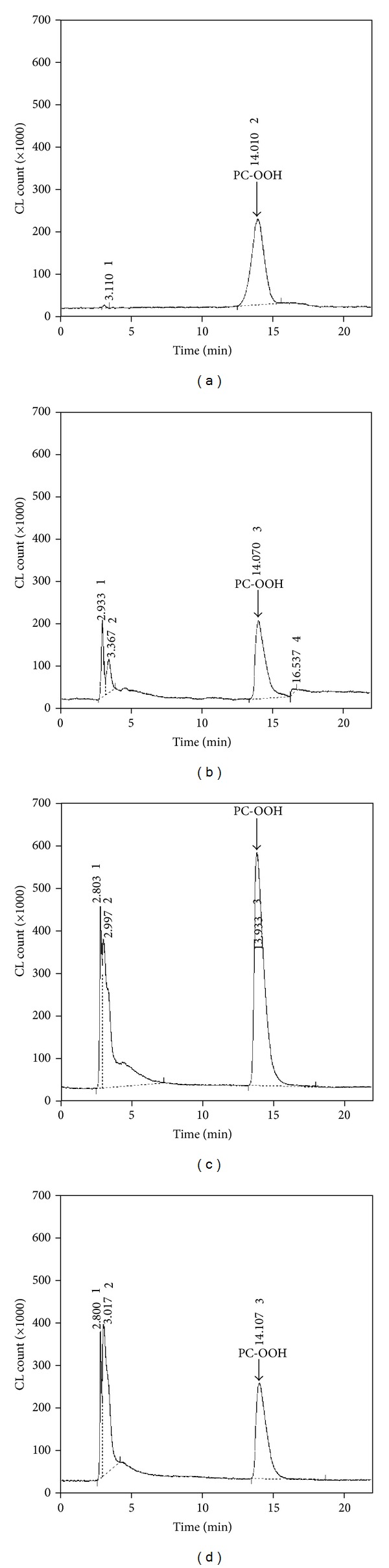
Representative chromatograms of the PC-OOH standard (402.8 pmol) (a), hepatic lipid extracts for the control (CON) (b), ethanol-treated (ALC) (c), and ethanol + curcumin-treated (CUR) (d) groups.

A photoirradiation method was used to synthesize a standard solution of PC-OOH for CL-HPLC analysis (Figure 2). Phosphatidylcholine (PC, from bovine heart, Sigma-Aldrich) was dissolved in methanol containing 0.01 mM methylene blue. The solution was exposed to a UV lamp (50 W) for 8 hr. Oxidant was cleaned up by using Supelclean LC-Si SPE tube (Supelco, Bellefonte, PA, USA) to remove methylene blue. The amount of peroxide in the standard solution was determined by method of the American Oil Chemist's Society [28]. As a result, the hydroperoxide concentration of the photooxidized PC was 19.1 *μ*mol hydroperoxide-O_2_ per g of PC.

### 2.4. Assay of PC-OOH

The PC-OOH level of total lipid extracts in a chloroform-methanol (2 : 1, v/v) solution was measured by using a CL-HPLC system [11]. The analytical system conditions were as follows: a Zorbax NH_2_ (4.6 × 250 mm, 5 *μ*m, Agilent Technologies, Wilmington, DE, USA) column was used for separation. The mobile phase consisted of 2-propanol-methanol-water (135 : 45 : 20, v/v/v), and the flow rate was 1 mL/min. The chemiluminescence analyzer (CLD-1100, Tohoku Electronic Industrial Co., Sendai, Japan) reaction temperature was 40°C, and the luminescence reagent was prepared by dissolving 10 mg/L of cytochrome c (from bovine heart, Sigma-Aldrich) and 2 mg/L of luminol (for chemiluminescence analysis, Wako pure chemical, Osaka, Japan) in 50 mM borate buffer (pH 10.0). This reagent was inserted into the sample chamber of the chemiluminescence analyzer by using a peristaltic pump at a flow rate of 1.0 mL/min [11, 13]. To calculate the recovery rate of this procedure, 3 different concentrations of the standard were produced as described above, and the peak areas of the prepreparative solution and postpreparative solutions were compared. The calculated recovery rate was 94.4% ± 4.7% (mean ± standard deviation) (SD)).

### 2.5. Statistical Analysis

All data are presented as mean ± SD values. Differences between the groups were calculated using Duncan's multiple range test (*P* < 0.05). All statistical analyses were performed using the Statistical Analysis Software (Statistics Analytical System Institute, Cary, NC, USA).

## 3. Results and Discussion

### 3.1. Body and Organ Weights

The body weights of ALC and CUR mice were significantly (*P* < 0.05) higher than those of CON mice (Table 1). This finding may be attributable to the effect of the extra calories contributed by excess ethanol consumption [29]. A similar pattern was observed for the spleen. We assume that reduced functioning of the liver caused by alcohol-related oxidative stress induced enlargement of the spleen [30]. Interestingly, the mean spleen weight of the CUR group was lower than that of the ALC group, but the difference was not significant. The kidney and brain weights of the alcohol-treated groups (ALC and CUR) were significantly (*P* < 0.05) lower than those of the CON group, suggesting that excessive alcohol consumption may affect the kidney and brain weights. However, the liver weights among all groups were not significantly different.

### 3.2. Lipid Content of the Liver and Brain

**Figure 3:**
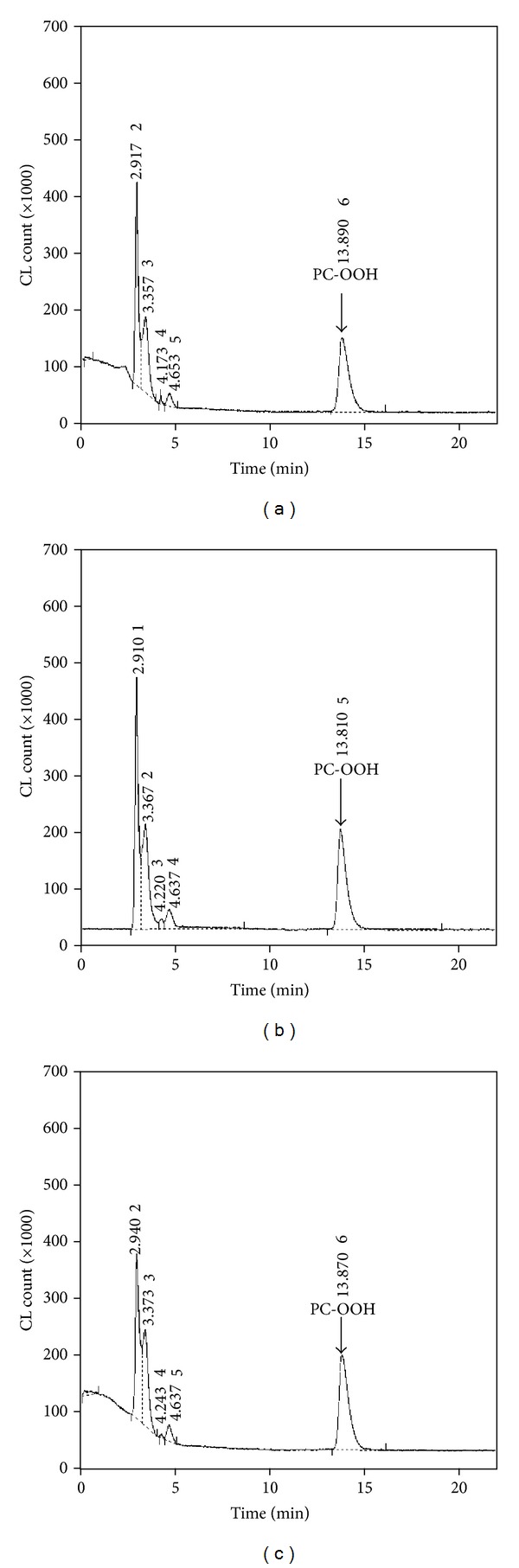
Representative chromatograms of brain lipid extracts for the CON (a), ALC (b), and CUR (c) groups.

The total lipid contents of the livers and brains of the three groups of mice are shown in Table 2. It was shown that hepatic total lipid and TG levels in the ALC group were significantly (*P* < 0.05) higher than the CON group. These results suggest that an alcohol-treated mouse develops fatty liver due to excessive alcohol consumption [31]. Although the lipid content of the livers of both ethanol-treated groups was high, the content in the CUR group was slightly lower than that of the ALC group, suggesting that steady consumption of curcumin inhibits the development of fatty liver to some extent. The most remarkable findings were that the hepatic TG and TC levels in the CUR group were significantly (*P* < 0.05) lower than those of the ALC group. This result suggests that daily consumption of even a small amount of curcumin may inhibit the development of alcohol-induced fatty liver and regulate hepatic cholesterol content. However, further investigation of these effects is required. No significant differences were observed between the brain lipid content (*P* < 0.05) of each group (Figure 3). It was thought that dietary 20% ethanol during 12 weeks did not affect the brain lipid contents in this study.

### 3.3. PC-OOH Content of the Liver and Brain

The hepatic PC-OOH content per total lipids did not differ between each group (data not shown). However, the content in the ALC group was significantly (*P* < 0.05) higher than in the CON and CUR groups, when the data were expressed by the modified lipid concentration, which was extracted by hepatic TG and TC concentrations from the total lipid (Table 3), because those lipids are not concerned with the oxidation of phospholipids. This finding may be attributable to liver damage induced by alcohol-related oxidative stress [2]. The decreased hepatic PC-OOH level in the CUR group indicates that curcumin consumption effectively protected the liver against alcohol-related oxidative stress. 

Several *in vitro* data indicate that curcumin has antioxidant, antiinflammatory, and anti-amyloid activities [17–20]. Furthermore, studies in animal models of Alzheimer's disease reviewed a direct effect of curcumin in decreasing the amyloid pathology of Alzheimer's disease [17]. However, the brain PC-OOH concentration did not significantly differ among all, suggesting that the dosage of alcohol could not increase a lipid peroxide level in brain and the reduced brain weight was not attributable to cell destruction caused by alcohol consumption. The differential effects of alcohol on the liver and brain PC-OOH levels might be the different mechanism of alcohol-related liver or brain disease because brain is affected in the final phase of alcohol-related disease while liver is affected in the primary phase [32]. Furthermore, brain damage by excessive alcohol consumption is linked to various conditions, such as alcohol-related liver diseases and thiamine deficiency. The mechanisms have not yet been clearly elucidated [32]. Therefore, further studies will be required to determine the effects of long-term treatment and behavioral tasks to better define the protective effect of curcumin on alcohol-related brain disease.

## 4. Conclusion

In this study, we demonstrated the effects of curcumin on alcohol-induced oxidative stress in liver but not in brain in mice treated with 20% ethanol. After 12 weeks, the hepatic PC-OOH level in the ALC group was observed to be higher than in the CON and ALC groups. This finding suggests that daily curcumin consumption is effective in protecting the liver against alcohol-related oxidative stress and indicates the possible role of hepatic lipid composition in altering the effect of excessive alcohol consumption. Therefore, further study is required for a detailed lipid mechanism of curcumin in mice fed an alcohol. In contrast, no significant difference was observed in the PC-OOH concentration among all groups. Therefore, additional studies may be required to investigate the effects of curcumin on the brains of alcohol-treated mice.

## Figures and Tables

**Table 1:** Organ weights.

	CON	ALC	CUR
Final body weight (g)	20.8 ± 2.2^b^	26.6 ± 1.7^a^	25.2 ± 1.1^a^
Liver (g/kg body weight)	32.6 ± 3.3	34.6 ± 1.3	34.4 ± 1.2
Kidney (g/kg body weight)	17.0 ± 0.8^a^	14.4 ± 0.8^b^	14.8 ± 0.8^b^
Spleen (g/kg body weight)	2.65 ± 0.37^b^	3.66 ± 0.48^a^	3.23 ± 0.31^a^
Brain (g/kg body weight)	16.6 ± 2.0^a^	12.7 ± 1.3^b^	13.7 ± 0.7^b^

All values are means ± SD. ^a-b^indicates significant differences between values in the same row (*P* < 0.05).

CON: high-fat diet; ALC: high-fat diet and alcohol treated; CUR: curcumin containing high-fat diet and alcohol treated.

**Table 2:** Concentrations of total lipids, triglyceride, and total cholesterol in the livers and brains.

(mg/100 mg wet tissue)		CON^1^	ALC	CUR
	Total lipid	8.72 ± 2.19^b^	16.8 ± 5.5^a^	15.2 ± 3.9^ab^
Liver	Triglyceride	5.94 ± 0.56^c^	12.5 ± 0.6^a^	9.34 ± 0.87^b^
	Total cholesterol	3.15 ± 0.92^b^	5.06 ± 2.12^a^	3.17 ± 0.78^b^

	Total lipid	11.8 ± 2.3	12.9 ± 1.7	13.2 ± 2.0
Brain	Triglyceride	8.34 ± 0.77	9.99 ± 2.22	8.73 ± 1.30
	Total cholesterol	1.16 ± 0.45	0.96 ± 0.35	1.03 ± 0.61

All values are means ± SD. ^a–c^indicates significant differences between values in the same row (*P* < 0.05).

CON: high-fat diet; ALC: high-fat diet and alcohol treated; CUR: curcumin containing high-fat diet and alcohol treated.

**Table 3:** The phosphatidylcholine hydroperoxide (PC-OOH) level of the liver and brain in mice.

(pmol/g lipid)		CON	ALC	CUR
PC-OOH*	Liver	286 ± 35^b^	366 ± 58^a^	157 ± 44^c^
	Brain	44.4 ± 12.9	53.0 ± 20.0	40.8 ± 5.9

All values are means ± SD. *was calculated by total lipid, which was extracted with triglyceride and total cholesterol concentrations. ^a–c^indicates significant differences between values in the same row (*P* < 0.05).

CON: high-fat diet; ALC: high-fat diet and alcohol treated; CUR: curcumin containing high-fat diet and alcohol treated.

## References

[1] Albano E (2006). Alcohol, oxidative stress and free radical damage. *Proceedings of the Nutrition Society*.

[2] Mann RE, Smart RG, Govoni R (2003). The epidemiology of alcoholic liver disease. *Alcohol Research and Health*.

[3] Moriya K, Nakagawa K, Santa T (2001). Oxidative stress in the absence of inflammation in a mouse model for hepatitis C virus-associated hepatocarcinogenesis. *Cancer Research*.

[4] Lee K, Møller L, Hardt F, Haubek A, Jensen E (1979). Alcohol-induced brain damage and liver damage in young males. *Lancet*.

[5] Soxton J, Munro CA, Butters MA, Schramke C, McNeil MA (2000). Alcohol, dementia, and alzheimer’s disease: comparison of neuropsychological profiles. *Journal of Geriatric Psychiatry Neurology*.

[6] Harper C, Matsumoto I (2005). Ethanol and brain damage. *Current Opinion Pharmacology*.

[7] Lovell MA, Markesbery WR (2007). Oxidative damage in mild cognitive impairment and early Alzheimer’s disease. *Journal of Neuroscience Research*.

[8] Slater TF (1984). Free-radical mechanisms in tissue injury. *Biochemistry Journal*.

[9] Slater TF (1987). Free radicals and tissue injury: fact and fiction. *The British Journal of Cancer*.

[10] Machlin LJ, Bendich A (1987). Free radical tissue damage: protective role of antioxidant nutrients. *The FASEB Journal*.

[11] Miyazawa T, Suzuki T, Fujimoto K, Yasuda K (1992). Chemiluminescent simultaneous determination of phosphatidylcholine hydroperoxide and phosphatidylethanolamine hydroperoxide in the liver and brain of the rat. *Journal of Lipid Research*.

[12] Suzuki M, Fukuhara K, Unno M (2000). Correlation between plasma and hepatic phosphatidylcholine hydroperoxide, energy charge, and total glutathione content in ischemia reperfusion injury of rat liver. *Hepato-Gastroenterology*.

[13] Adachi J, Matsushita S, Yoshioka N (2004). Plasma phosphatidylcholine hydroperoxide as a new marker of oxidative stress in alcoholic patients. *Journal of Lipid Research*.

[14] Aggarwal BB, Shishodia S (2004). Suppression of the nuclear factor-*κ*B activation pathway by spice-derived phytochemicals: reasoning for seasoning. *Annals of the New York Academy of Sciences*.

[15] Kohli K, Ali J, Ansari MJ, Raheman Z (2005). Curcumin: a natural antiinflammatory agent. *Indian Journal of Pharmacology*.

[16] Hatcher H, Planalp R, Cho J, Torti FM, Torti SV (2008). Curcumin: from ancient medicine to current clinical trials. *Cellular and Molecular Life Sciences*.

[17] Ringman JM, Frautschy SA, Cole GM, Masterman DL, Cummings JL (2005). A potential role of the curry spice curcumin in Alzheimer’s disease. *Current Alzheimer Research*.

[18] Balasubramanyam M, Koteswari AA, Kumar RS, Monickaraj SF, Maheswari JU, Mohan V (2003). Curcumin-induced inhibition of cellular reactive oxygen species generation: novel therapeutic implications. *Journal of Biosciences*.

[19] Lim GP, Chu T, Yang F, Beech W, Frautschy SA, Cole GM (2001). The curry spice curcumin reduces oxidative damage and amyloid pathology in an Alzheimer transgenic mouse. *Journal of Neuroscience*.

[20] Kowluru RA, Kanwar M (2007). Effects of curcumin on retinal oxidative stress and inflammation in diabetes. *Nutrition and Metabolism*.

[21] Samuhasaneeto S, Thong-Ngam D, Kulaputana O, Suyasunanont D, Klaikeaw N (2009). Curcumin decreased oxidative stress, inhibited NF-*κ*B activation, and improved liver pathology in ethanol-induced liver injury in rats. *Journal of Biomedicine and Biotechnology*.

[22] Bachmanov AA, Reed DR, Beauchamp GK, Tordoff MG (2002). Food intake, water intake, and drinking spout side preference of 28 mouse strains. *Behavior Genetics*.

[23] Rukkumani R, Balasubashini MS, Menon VP (2003). Protective effects of curcumin and photo-irradiated curcumin on circulatory lipids and lipid peroxidation products in alcohol and polyunsaturated fatty acid-induced toxicity. *Phytotherapy Research*.

[24] Lim GP, Chu T, Yang F, Beech W, Frautschy SA, Cole GM (2001). The curry spice curcumin reduces oxidative damage and amyloid pathology in an Alzheimer transgenic mouse. *Journal of Neuroscience*.

[25] Ringman JM, Frautschy SA, Cole GM, Masterman DL, Cummings JL (2005). A potential role of the curry spice curcumin in Alzheimer’s disease. *Current Alzheimer Research*.

[26] Polavarapu R, Spitz DR, Sim JE (1998). Increased lipid peroxidation and impaired antioxidant enzyme function is associated with pathological liver injury in experimental alcoholic liver disease in rats fed diets high in corn oil and fish oil. *Hepatology*.

[27] Folch J, Lees M, Sloane-Stanley GH (1957). A simple method for the isolation and purification of total lipides from animal tissues. *The Journal of Biological Chemistry*.

[28] (1964). *Official and Tentative Methods of AOCS, Cd 8-53*.

[29] Suter PM, Häsler E, Vetter W (1997). Effects of alcohol on energy metabolism and body weight regulation: is alcohol a risk factor for obesity?. *Nutrition Reviews*.

[30] Pazo AL, Godfrey EM, Bowles KM (2009). Splenomegaly: invetigation, diagnosis and management. *Blood Reviews*.

[31] Lieber CS (1975). Liver disease and alcohol: fatty liver, alcoholic hepatitis, cirrhosis, and their interrelationships. *Annals of the New York Academy of Sciences*.

[32] Butterworth RF (1995). Pathophysiology of alcoholic brain damage: synergistic effects of ethanol, thiamine deficiency and alcoholic liver disease. *Metabolic Brain Disease*.

